# Task Offloading Strategy for Unmanned Aerial Vehicle Power Inspection Based on Deep Reinforcement Learning

**DOI:** 10.3390/s24072070

**Published:** 2024-03-24

**Authors:** Wei Zhuang, Fanan Xing, Yuhang Lu

**Affiliations:** School of Computer Science, Nanjing University of Information Science and Technology, Nanjing 210044, China

**Keywords:** multiple UAVs, mobile edge computing, deep reinforcement learning, power inspection, offloading strategy, electric power IoT

## Abstract

With the ongoing advancement of electric power Internet of Things (IoT), traditional power inspection methods face challenges such as low efficiency and high risk. Unmanned aerial vehicles (UAVs) have emerged as a more efficient solution for inspecting power facilities due to their high maneuverability, excellent line-of-sight communication capabilities, and strong adaptability. However, UAVs typically grapple with limited computational power and energy resources, which constrain their effectiveness in handling computationally intensive and latency-sensitive inspection tasks. In response to this issue, we propose a UAV task offloading strategy based on deep reinforcement learning (DRL), which is designed for power inspection scenarios consisting of mobile edge computing (MEC) servers and multiple UAVs. Firstly, we propose an innovative UAV-Edge server collaborative computing architecture to fully exploit the mobility of UAVs and the high-performance computing capabilities of MEC servers. Secondly, we established a computational model concerning energy consumption and task processing latency in the UAV power inspection system, enhancing our understanding of the trade-offs involved in UAV offloading strategies. Finally, we formalize the task offloading problem as a multi-objective optimization issue and simultaneously model it as a Markov Decision Process (MDP). Subsequently, we proposed a task offloading algorithm based on a Deep Deterministic Policy Gradient (OTDDPG) to obtain the optimal task offloading strategy for UAVs. The simulation results demonstrated that this approach outperforms baseline methods with significant improvements in task processing latency and energy consumption.

## 1. Introduction

Due to the ongoing advancement of electric power IoT, ensuring the stable operation of the power system has become vital for socioeconomic progress [1,2,3]. However, traditional power inspections primarily rely on manual surveillance and periodic maintenance, resulting in issues such as long inspection cycles, low efficiency, and high costs. Additionally, the widespread distribution of power facilities and adverse environmental conditions in some areas make traditional inspection methods inadequate for meeting practical requirements [4,5].

Over the past few years, the rapid advancement of UAVs has brought about innovative solutions for power inspection [6]. UAVs are characterized by high maneuverability, ease of deployment, excellent line-of-sight communication, and strong adaptability to complex environments [7]. These characteristics enable UAVs to conduct more flexible monitoring and inspection of electrical facilities, with broader coverage and increased efficiency. Moreover, UAVs are equipped with onboard cameras, electro-optical observation systems, infrared thermal imaging, laser radar, and other relevant detection sensors, which can avoid direct contact between inspection personnel and high-voltage electrical facilities, thus reducing safety risks [8]. In UAV power inspections, due to limitations in computational power and battery capacity, inspection tasks are typically offloaded to a central cloud for processing [9]. For computationally intensive tasks, Mobile Cloud Computing (MCC) can assist performance-constrained UAVs in handling these computationally demanding missions and returning the results, significantly alleviating the computational burden on UAVs [10]. However, in traditional cloud-centric computing models, the considerable distance between the central cloud server and UAVs may lead to substantial data transmission delays, which may not be conducive to timely fault information retrieval by inspection personnel, especially for latency-sensitive inspection tasks [11].

Mobile Edge Computing (MEC) is primarily focused on bringing data processing and computational resources in proximity to data sources and end-user devices, thereby reducing data transmission latency and network congestion [12]. Consequently, MEC effectively addresses the issues associated with traditional centralized cloud systems by providing computational resources at the network edge, such as deploying edge servers near base stations. This approach allows for the proximity of computation and data storage functions to inspection sites, resulting in lower latency and higher real-time capabilities [13,14]. The application of MEC technology enables UAVs to access real-time data, perform efficient data processing, and make decisions during power inspections, thereby enhancing the accuracy and efficiency of inspection tasks [15]. 

During the UAV power inspection process, MEC effectively addresses the limitations in UAV battery capacity and computational capabilities [16]. However, an improper offloading strategy can lead to performance degradation, increased energy consumption, inefficient task execution, and even affect system stability in the UAV power inspection system. Therefore, we propose an optimization algorithm for UAV power inspection task offloading strategies based on Deep Deterministic Policy Gradients (OTDDPGs). Firstly, in the context of UAV power inspection scenarios utilizing MEC, we propose an innovative UAV-Edge server collaborative computing architecture. The core concept of this architecture is to exploit UAVs’ mobility and the powerful computing capabilities of MEC servers to boost the efficiency and performance of UAV power grid inspections. Secondly, based on the computation offloading mode of UAVs (where UAVs offload some computational tasks to MEC servers while executing the remaining task locally) and the inspection mode, we established a computational model for the system energy consumption and the latency. While ensuring the accuracy and timeliness of inspection tasks, decisions are made by balancing factors such as specific task requirements, the UAV’s resource status, and the communication environment. Finally, we formalize the task offloading problem as an optimization problem with multiple objectives, aiming to minimize the combined impact of system energy consumption and task processing latency. We formulate this as a Markov Decision Process (MDP) and propose the OTDDPG algorithm to address the optimization problem of UAV task offloading in a continuous action space, ultimately obtaining the optimal offloading strategy. The primary contributions of this paper can be summarized as follows:

- Regarding the power inspection scenario consisting of MEC servers and multiple UAVs, an innovative UAV-Edge server collaborative computing architecture is proposed. This architecture aims to exploit the mobility of UAVs and the high-performance computing capability of MEC servers to enhance the efficiency and performance of power inspection.
- A computational model for system energy consumption and task processing latency has been established based on the computation offloading mode and inspection mode of UAVs. This model takes into account various critical factors, such as specific task requirements (e.g., time constraints on task execution), UAV resource limitations (e.g., computational capacity and battery life), and communication environment (e.g., channel state information), to assist us in better understanding the trade-off issues in UAV offloading strategies.
- We formalize the task offloading problem as an optimization problem with multiple objectives, aiming to minimize the combined impact of system energy consumption and latency. Simultaneously, we formulate this as an MDP and propose the OTDDPG algorithm to derive the best task offloading strategy for UAVs. This algorithm demonstrates a high adaptability to continuous action spaces and efficiently searches for the optimal strategy.

## 2. Related Works

In recent years, both domestically and internationally, numerous research efforts have been conducted on task offloading problems targeting various optimization objectives. By formulating sensible offloading strategies, it becomes possible to transfer tasks to resource-rich edge servers, thereby achieving objectives such as reducing latency and conserving system energy consumption.

Ref. [17] initially proposed general guidelines for offloading decisions aimed at reducing energy consumption. However, it assumed that the channel state remains constant. Thus, Ref. [18] further introduced optimal binary offloading decisions. Ref. [19] employed deep learning to address the heterogeneity of task requirements in various offloading modes, significantly reducing the algorithm complexity. Ref. [20] discusses the role of mobile edge computing (MEC) in reducing data transmission latency between mobile devices and servers. It proposed task offloading strategies based on Particle Swarm Optimization (PSO) and Quantum PSO to address the limited resources of MEC servers and the inability to handle all mobile computing tasks. The extensive simulation results demonstrated the superior performance of the proposed algorithms in terms of system energy consumption, task completion time, and execution time compared to other state-of-the-art strategies. Ref. [21] presents a multi-UAV power inspection offloading strategy based on game theory and reinforcement learning. They employed a distributed algorithm to discover the Nash equilibrium solution for both sides of the game while minimizing the combined cost function, which incorporates energy consumption and latency. This achieves the offloading of transmission line inspection tasks to appropriate edge servers. Ref. [22] proposed a novel joint optimization model aimed at coordinating the charging operations of multiple UAVs acting as aerial base stations. By optimizing the allocation and trajectory of charging stations, the model seeks to extend the flight time of UAVs and minimize the overall energy consumption. Compared to traditional greedy heuristic methods, the model can reduce energy usage by 9.1%. Ref. [23] designed an adaptive K algorithm for multi-UAV scenarios to reduce problem complexity through the collaborative optimization of UAV communication time resources using convex optimization algorithms to attain the optimal offloading strategy. Ref. [24] discusses the deployment of UAV-assisted MEC networks in a short time frame and proposed a deep reinforcement learning model for end-to-end learning and optimization of task offloading and UAV trajectory control. The model aimed to optimize multiple objectives by controlling the proportion of task offloading and UAV trajectory, maximizing the number of tasks processed while minimizing energy and time consumption. The experimental results demonstrated that the proposed model outperforms the existing methods. Ref. [25] framed the task offloading problem for power grid inspections in a UAV edge computing environment as an optimization problem to minimize latency under computational and communication resource constraints. The data indicated that DQN effectively reduced the UAV detection latency and enhanced detection efficiency. Ref. [26] addressed the computation offloading and resource allocation challenges in a multi-user, multi-UAV mobile edge cloud computing system. It introduced an efficient and scalable model for resource allocation and computation offloading. The model utilizes multi-tier mobile edge computing (MEC) technology and software-defined networking (SDN) to manage core network traffic. The experimental results demonstrated significant performance improvements in both time and energy aspects. In Ref. [27], a fastest descent resource allocation algorithm was introduced. An improved multi-objective evolutionary algorithm was developed to dynamically adjust neighborhood sizes and crossover distribution parameters. Through simulation experiments, it was demonstrated that this approach significantly reduced service latency and energy consumption compared to other algorithms. Ref. [28] outlined a scenario in which a lone ground station offers computational offloading services to an individual UAV. The UAV divides tasks into local computation and ground station offloading while in flight. To mitigate UAVs’ energy consumption, a progressive convex approximation approach was employed to jointly optimize both the UAVs’ flight path and their position allocation, resulting in a suboptimal solution with minimal UAV energy consumption. The experimental results demonstrated that this approach effectively saved a significant amount of energy. In Ref. [29], a multi-UAV collaborative task allocation strategy was developed based on ant colony optimization methods in the context of a collaborative battlefield scenario. Through an experimental analysis, the proposed multi-UAV collaborative task allocation algorithm’s rationality and effectiveness were validated to meet the requirements of collaborative combat missions. Ref. [30] explores a novel MEC application scenario. It jointly optimizes UAV trajectories and computation offloading schedules under constraints on the maximum UAV speed and the computational capabilities of the Ground Base Stations (GBSs) to minimize the task completion time. Simultaneously, they proposed an effective method for obtaining suboptimal solutions to the problem. The data demonstrated that this approach led to a substantial reduction in system latency. However, most of the aforementioned offloading methods lack flexibility and cannot adapt to real-time changes in task requirements and communication environments.

Building upon this, we propose a task offloading strategy for UAV power inspections based on DRL. Unlike previous research, this offloading strategy focuses on real-time and dynamic aspects to maximize the optimization effect on system energy consumption and latency. Comprehensively considering the UAV’s resource status, communication environment, and task requirements, we constructed a novel task offloading model to support the efficiency of UAV power inspection operations.

## 3. System Model and Problem Formulation

**Figure 1:**
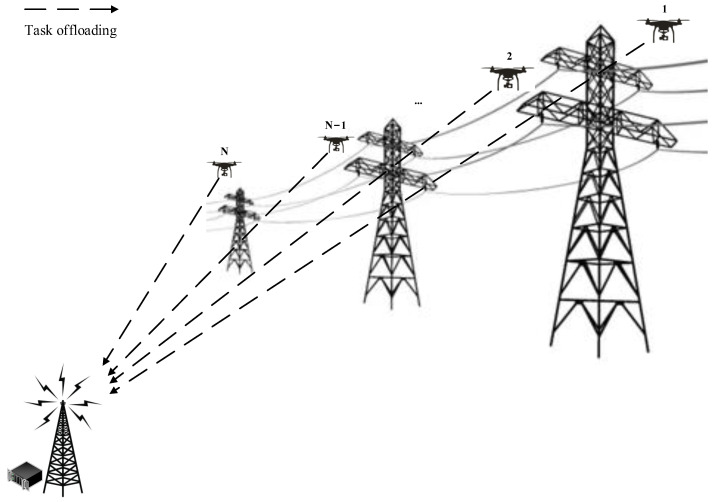
MEC-based UAV power inspection system model.

Our MEC-based UAV power inspection system is shown in Figure 1. The system primarily consists of a multi-antenna base station (BS) integrated with an MEC server, positioned at a fixed location, and N single-antenna unmanned aerial vehicles (UAVs). The entire system time is divided into equal-length consecutive time slots using the Time Division Multiple Access (TDMA) protocol [31]. We adopt Orthogonal Frequency Division Multiplexing (OFDM) as the channel access method. OFDM technology effectively supports simultaneous communication between MEC servers and multiple UAVs by improving spectrum efficiency, resisting multi-path fading, enabling flexible resource allocation, accommodating multiple user access, and providing robust interference resistance. The MEC server delivers computing services to all UAVs, which, following a partial offloading mode, can execute some computing tasks locally and offload the remaining tasks to the MEC server for execution. The symbols for the variables are shown in Table 1.

### 3.1. Communication Model

Since UAV inspections involve continuous actions, the system time ‘T’ is partitioned into ‘I’ equal continuous time slots. Taking into account the mobility of UAVs and the influence of the communication environment, wireless channel gains between different time slots may vary. However, over short time scales, significant changes in path loss or alterations in multipath effects may not occur. Therefore, it is reasonable to approximate the channel gains as constant within the same time slot. The MEC server was deployed at the base station for power management and was equipped with a high-speed multi-core CPU, providing ample computing resources. As a result, the computational energy consumption of the server was not considered. The UAVs were equipped with on-board cameras, electro-optical observation systems, infrared thermal imagers, lidars, and other sensors for real-time collection of information about power facilities. In each time slot, all UAVs first flew from their starting positions to their designated ending positions within a specified time frame. Then, at the ending positions, they employed OFDM technology to transfer some of the tasks for execution to the MEC servers, and the remaining part was executed on the UAV. Throughout entire inspection process, the UAVs maintained an altitude ‘H’ and the ending position of the previous time slot served as the starting position for the next time slot. In three-dimensional space, the fixed position of MEC was defined in terms of coordinates as $Q_{mec}=(x_{mec},y_{mec,}0)$. The starting position of the k-th UAV in the i-th time slot was defined as $Q_{uav,k}(i-1)=(x_{uav,k}(i-1),y_{uav,k}(i-1),H),i\in \{1,\cdots ,I\},k\in \{1,\cdots ,N\}$, and the ending position was defined as $Q_{uav,k}(i)=(x_{uav,k}(i),y_{uav,k}(i),H)$. The channel gain of the line-of-sight link between the k-th UAV and the MEC server in the i-th time slot is given by

(1) $$h_{k}(i)=\beta_{0}d_{k}^{-2}(i)=\frac{\beta_{0}}{{\left\|Q_{uav,k}(i)-Q_{mec}\right\|}^{2}+H^{2}}$$

where $\beta_{0}$ represents the channel gain when the reference distance is 1 m. Due to obstacles blocking the path, the uplink transmission rate of the k-th UAV in the i-th time slot is

(2) $$r_{k}(i)=B_{0}\log_{2}(1+\frac{P_{uav}h_{k}(i)}{\sigma^{2}+c_{k}(i)P_{NLOS}})$$

where $B_{0}$ represents the channel bandwidth allocated to each UAV, assuming that the transmission power of all UAVs is $P_{uav}$, $\sigma^{2}$ represents the noise power, $P_{NLOS}$ represents the propagation loss, and $c_{k}(i)\in \{0,1\}$ represents the presence or absence of obstruction between the k-th UAV and the MEC server during the i-th time slot (0 indicates no blockage, 1 indicates a blockage) [32].

### 3.2. Delay Model

The task processing delay mainly includes the computation delay of UAVs executing tasks locally, the transmission delay of UAVs sending task data to the MEC server, and the computation delay at the MEC server.

As a UAV performs part of the inspection computation task locally, the computation delay of the k-th UAV executing tasks locally during the i-th time slot is defined as

(3) $$t_{uav,k}(i)=\frac{(1-R_{k}(i))D_{k}(i)s}{f_{uav}}$$

where $D_{k}(i)$ denotes the size of the data used for the computations for the k-th UAV during the i-th time slot; $R_{k}(i)\in [0,1]$ represents the task proportion of the k-th UAV unloaded to the MEC server in the i-th time slot; ${1-R}_{k}(i)$ indicates the fraction of the k-th UAV tasks that are performed locally in the i-th time slot; and s denotes the CPU cycles necessary for processing each data byte, assuming that the CPU computing frequency $f_{uav}$ is the same for all UAVs.

The uplink transmission rate determines the transmission delay for UAVs as they send the remaining inspection tasks to the MEC server. Therefore, the transmission delay for the k-th UAV sending task data to the MEC server in the i-th time slot is given by

(4) $$t_{tr,k}(i)=\frac{R_{k}(i)D_{k}(i)}{r_{k}(i)}$$

When offloading the remaining inspection tasks from the UAV to the MEC server for execution, the computation delay for the MEC server to deliver computing services to the k-th UAV in the i-th time slot can be described as follows:

(5) $$t_{mec,k}(i)=\frac{R_{k}(i)D_{k}(i)s}{f_{mec}}$$

assuming that the CPU computing frequency of the MEC sever is $f_{mec}$.

In summary, the UAV power inspection system’s maximum task processing delay can be represented as

(6) $$t_{total}(i)=\underset{k\in \{1,\cdots ,N\}}{\max}\left(t_{uav,k}(i),t_{tr,k}(i)+t_{mec,k}(i)\right)$$

### 3.3. Energy Model

System energy consumption includes the flying energy consumption of the UAVs, the computational energy consumption when the UAVs perform tasks locally, and the transmission energy consumption when the UAVs offload tasks to the MEC servers. 

During the i-th time slot, the UAV inspection process requires flying from the starting location to the ending location:

(7) $$Q_{uav,k}(i)=(x_{uav,k}(i-1){+v}_{k}(i-1)\tau \cos\beta_{k}(i-1),y_{uav,k}(i-1){+v}_{k}(i-1)\tau \sin\beta_{k}(i-1),H)$$

where $v_{k}(i-1)\in (0,v_{max})$ is the flight speed of the k-th UAV in time slot i-1, $\beta_{k}(i-1)\in (0,2\pi )$ is the flight angle of the k-th UAV in time slot i−1, and τ represents the flight time of the UAV. The flying energy consumption of the UAV for performing inspection tasks can be represented as

(8) $$e_{fly,k}(i)=\frac{1}{2}M\tau {\left\|v_{k}(i)\right\|}^{2}$$

where M is associated with the UAV’s payload [33]. 

When the UAV locally performs a portion of the inspection computing tasks, the processor unit and its corresponding computing unit in the chip architecture consume energy during task execution. Therefore, the computational energy consumption depends on the impact factors of the chip structure on the UAV’s CPU processing. The computational energy consumption of the k-th UAV when performing tasks locally in the i-th time slot is

(9) $$e_{uav,k}(i)=k_{uav}f_{uav}^{3}t_{uav,k}(i)=k_{uav}f_{uav}^{2}(1-R_{k}(i))D_{k}(i)s$$

assuming that the energy coefficients of the chip architectures of all UAVs is $k_{uav}$.

The transmission energy consumption when the k-th UAV offload tasks to the MEC servers in the i-th time slot is

(10) $$e_{tr,k}(i)=P_{uav}t_{tr,k}(i)=P_{uav}\frac{R_{k}(i)D_{k}(i)}{r_{k}(i)}$$

In summary, the UAV power inspection system’s total energy consumption can be represented as

(11) $$e_{total}(i)=\sum_{k=1}^{N}{(e}_{uav,k}(i)+e_{tr,k}(i)+e_{fly,k}(i))$$

### 3.4. Problem Formulation

To ensure real-time performance and adaptability and by taking into account the resource status of UAVs, communication environment, and task requirements, with a weighted sum that minimizes system energy consumption and delay, the optimization problem of UAV task offloading strategy can be modeled as

(12) $$\underset{t_{\mathrm{total}}(i),e_{\mathrm{total}}(i)}{\min}\sum_{i=1}^{I}\left[\omega t_{total}(i)+\varpi e_{total}(i)\right]\;s.t.\;C1:\;0\leq R_{k}(i)\leq 1,\forall i,k\;C2:t_{total}(i)+\tau \leq \frac{T}{I},\forall i\;C3:Q_{mec},Q_{uav,k}(i)\leq \{\;X_{size},Y_{size}\},\forall i,k\;C4:v_{k}(i)\in [0,v_{max}],\beta_{k}(i)\in [0,2\pi ],\forall i,k\;C5:\;c_{k}(i)\in \{0,1\},\forall i,k\;C6:\;\sum_{i=1}^{I}{(e}_{uav,k}(i)+e_{tr,k}(i)+e_{fly,k}(i))\leq E,\forall k\;C7:\;\sum_{i=1}^{I}\sum_{k=1}^{N}D_{k}(i)=D$$

where ω is the delay weight parameter, ϖ represents the energy consumption weight parameter, $\omega ,\varpi \in \left[0,1\right],\;and\;\omega +\varpi =1$. The use of a weighted function enhances flexibility, allowing the use of different weight parameters for different computing tasks based on the current system state. For computing tasks with high delay requirements, the delay weight parameter can be increased. If the UAV battery is at a low level, priority is given to the energy consumption parameter, aiming to conserve more energy. C1 represents the range of the offloading ratio values for computing tasks. C2 represents the time constraints for each time slot. C3 represents the location constraints between the MEC server and UAVs. C4 represents the constraints on UAV flight speed and angles. C5 represents the congestion constraint of the wireless channel between the drone and the MEC server, aiding the system in adapting to varying levels of congestion and communication demands. C6 represents the battery capacity constraint of the UAV, and C7 represents the total task size for each episode of inspection.

## 4. Algorithm Design

This section introduces the OTDDPG algorithm for addressing the task offloading optimization problem described above. This method effectively supports optimization problems with a continuous action space for UAV mobility, while reducing system energy consumption and latency.

### 4.1. Proposed OTDDPG Method

DDPG is an algorithm that was introduced by the Google DeepMind team. It is used to output deterministic actions, addressing the drawback of the Actor–Critic neural network that having correlations between parameter updates before and after leads to a limited perspective on problems [34]. It also addresses the limitation of DQNs, which cannot be used for continuous actions. DDPG inherits the deterministic policy from Deterministic Policy Gradients (DPGs). The agent decides deterministic actions based on the state, and DDPG employs deep neural networks to improve the decision function’s capacity for fitting.

**Figure 2:**
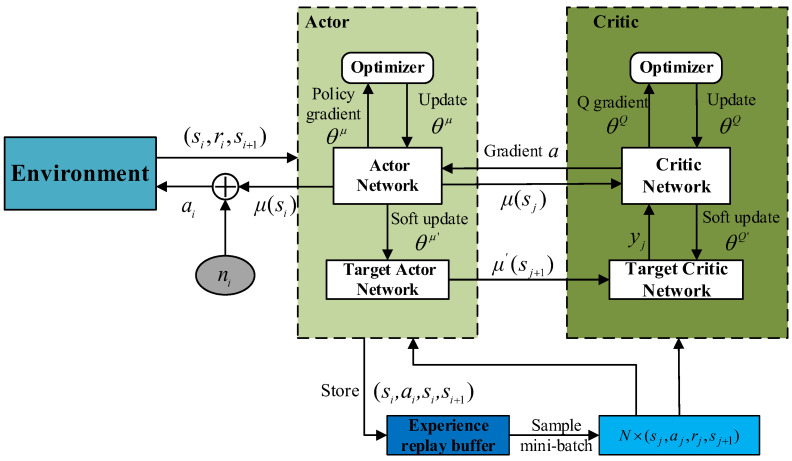
DDPG algorithm architecture.

Figure 2 depicts the DDPG algorithm architecture. To address the instability in Q-value learning within a single neural network, DDPG adopts a dual-network architecture. It creates two neural networks for the Actor and Critic, respectively, and employs a soft update approach for network updates to ensure more stable training. The Actor network is tasked with learning the policy and translating states into actions within the continuous action space. The Critic network assesses the performance of the Actor’s policy, estimating Q-values for state–action pairs and guiding the generation of actions in the next stage. An experience replay buffer stores the data generated by the agent during its interaction with the environment, addressing the issue of sample data correlations in traditional Actor–Critic algorithms.

Below, we formalize the task offloading problem as an MDP by defining states, actions, and rewards.

State: The state space describes critical information and features in the environment. In the context of the UAV power inspection system, the state space is defined by the resource status of N UAVs and environmental information. Thus, the system state in the i-th time slot is

(13) $$s_{i}=\{D_{remain}(i),Q_{mec},E_{1}^{remain}(i),\cdots ,E_{N}^{remain}(i),Q_{uav,1}(i),\cdots ,Q_{uav,N}(i),D_{1}(i),\cdots ,D_{N}(i),c_{1}(i),\cdots ,c_{N}(i)\}$$

where $D_{remain}(i)$ represents the total remaining computation task size, $Q_{mec}$ denotes the coordinates of the MEC server, $E_{N}^{remain}(i)$ is the N-th UAV’s remaining power in the i-th time slot, $Q_{uav,N}(i)$ denotes the N-th UAV’s coordinates in the i-th time slot, $D_{N}(i)$ denotes the size of the data used in the computations for the N-th UAV during the i-th time slot, and $c_{N}(i)$ indicates whether there is wireless channel congestion between the N-th UAV and the MEC server in the i-th time slot.

Action: The action space represents the range of decisions available to the agent. By observing the system’s state, the agent selects the task offloading rate for each UAV. Therefore, the system’s offloading decision action in the i-th time slot is

(14) $$a_{i}=\left\{R_{1}(i),R_{2}(i),\cdots ,R_{N}(i)\right\}$$

where $R_{N}(i)\in [0,1]$ is the task offloading ratio of the N-th UAV in the i-th time slot.

Reward: Given the previously mentioned system state space and action space, the reward function for the system in the i-th time slot is

(15) $$r_{i}=-(\omega t_{total}(i)+\varpi e_{total}(i)).$$

### 4.2. Learning Strategy

We initiated four neural networks: the Actor network with parameters $\theta^{\mu }$, the Critic network with parameters $\theta^{Q}$, the target Actor network with parameters $\theta^{\mu^{\prime }}$, and the target Critic network with parameters $\theta^{Q^{\prime }}$. We also initialized $\theta^{\mu }=\theta^{\mu^{\prime }},\;\theta^{Q}=\theta^{Q^{\prime }}$, and an experience replay buffer.

In the i-th time slot, to explore potential optimal strategies, OTDDPG selects actions $a_{i}=\mu (s_{i}|\theta^{\mu })+n_{i}$ by introducing random noise $n_{i}$, where $\mu (s_{i}|\theta^{\mu })$ is the deterministic policy function fitted by the Actor network. After all UAVs execute their actions, the agent can obtain the subsequent time slot state $s_{i+1}$ and immediate reward $r_{i}$, and store this state transition record $\left\{s_{i},a_{i},r_{i},s_{i+1}\right\}$ in the experience replay buffer.

When the data volume in the experience replay buffer reaches a sampleable condition, S samples are randomly drawn from the experience buffer using the mini-batch method, and one of the samples is denoted as $\left\{s_{j},a_{j},r_{j},s_{j+1}\right\}$. The subsequent time slot state $s_{j+1}$ and the action policy $\mu^{\prime }(s_{j+1}|\theta^{\mu^{\prime }})$ taken by the target Actor network are input into the target Critic network to calculate the state-action Q-value function for the subsequent time slot: $Q^{\prime }(s_{j+1},\mu^{\prime }(s_{j+1}|\theta^{\mu^{\prime }}))|\theta^{Q^{\prime }})$. Thus, the target value of the Q-value function for the current state can be obtained:

(16) $$y_{j}=r_{j}+\gamma Q^{\prime }(s_{j+1},\mu^{\prime }(s_{j+1}|\theta^{\mu^{\prime }})|\theta^{Q^{\prime }})$$

where $\gamma \in \left[0,1\right]$ is the discount factor. The current time slot state $s_{j}$ and action $a_{j}$ are input into the Critic network, which outputs the state-action Q-value function for the current time slot: $Q(s_{j},a_{j}|\theta^{Q})$. The goal of the Critic network is to learn the Q-value function. The network parameters are updated by minimizing the loss function through gradient descent on the Critic network:

(17) $$L(\theta^{Q})=\frac{1}{S}\sum_{j}{{(y}_{j}-Q(s_{j},a_{j}|\theta^{Q}))}^{2}$$

The relationship between the loss function of the Critic network and the Q-value function lies in optimizing the loss function to better approximate the true Q-value function. This provides a more accurate estimate for subsequent policy updates.

Combining the Q-value function, we can obtain the Actor network’s policy gradient while updating its parameter:

(18) $$\nabla_{\theta^{\mu }}J=\frac{1}{S}\sum_{j}\nabla_{a}Q(s,a|\theta^{Q}){|}_{s=s_{j},a=\mu (s_{j}|\theta^{\mu })}\nabla_{\theta^{\mu }}\mu (s|\theta^{\mu }){|}_{s=s_{j}}$$

where $\mu (s|\theta^{\mu })$ is the task offloading policy generated by inputting $s_{j}$ into the Actor network. After a certain number of training iterations, the parameter $\theta^{\mu^{\prime }}$ and the parameter $\theta^{Q^{\prime }}$ are updated by a soft update method as follows:

(19) $$\theta^{\mu^{\prime }}=\xi \theta^{\mu }+(1-\xi )\theta^{\mu^{\prime }}\;\theta^{Q^{\prime }}=\xi \theta^{Q}+(1-\xi )\theta^{Q^{\prime }}$$

where ξ is the soft update coefficient. After training is completed, the Actor network can execute the optimal task offloading policy for UAV computing tasks. The overall pseudocode for this algorithm is shown in Algorithm 1.
**Algorithm 1** OTDDPG Method.Initialize actor network $\mu (s|\theta^{\mu })$, and critic network $Q(s,a|\theta^{Q})$ with random weights $\theta^{\mu }$ and $\theta^{Q}$
 Initialize target networks $\mu^{\prime }$ and $Q^{\prime }$ with weights $\theta^{\mu^{\prime }}\leftarrow \theta^{\mu }$*,*$\theta^{Q^{\prime }}\leftarrow \theta^{Q}$
                                                     Initialize replay buffer B
 for episode = 1, M do     Initialize a random process n for action exploration;     Initialize ω*,*
ϖ, *D* and *E*;     Randomly initialize the state $s_{1}$;     for i = 1, I do         Select action $a_{i}=\mu (s_{i}|\theta^{\mu })+n_{i}$ according to the current policy and exploration and exploration noise         Enter state $s_{i}$ and action $a_{i}$ into the environment         for k = 1, N do             Initialize $D_{k}(i)$*,*$c_{k}(i)$*,*$v_{k}(i)$*,*$\beta_{k}(i)$
            Compute $t_{uav,k}(i),t_{tr,k}(i),t_{mec,k}(i)$*,*
$e_{fiy,k}(i),e_{uav,k}(i),e_{tr,k}(i)$;             Update $Q_{uav,k}(i)$*,*$E_{k}^{remain}(i)$;         end         Get $r_{i}=-(\omega t_{total}(i)+\varpi e_{total}(i))$
        Update $D_{remain}\left(i\right)$
        Obtain reward $r_{i}$ and new state $s_{i+1}$
        Store transition $\left(s_{i},a_{i},r_{i},s_{i+1}\right)$ in B
        Sample a random mini-batch of S transitions $\left(s_{j},a_{j},r_{j},s_{j+1}\right)$ from B
        Set $y_{j}=r_{j}+\gamma Q^{\prime }(s_{j+1},\mu^{\prime }(s_{j+1}|\theta^{\mu^{\prime }})|\theta^{Q^{\prime }})$
        Update critic by minimizing the loss: $L(\theta^{Q})=\frac{1}{S}\sum_{j}{{(y}_{j}-Q(s_{j},a_{j}|\theta^{Q}))}^{2}$
        Update the actor policy using the sampled gradient:                             $\nabla_{\theta^{\mu }}J=\frac{1}{S}\sum_{j}\nabla_{a}Q(s,a|\theta^{Q}){|}_{s=s_{j},a=\mu (s_{j}|\theta^{\mu })}\nabla_{\theta^{\mu }}\mu (s|\theta^{\mu }){|}_{s=s_{j}}$
        Update the target networks:                                                         $\theta^{\mu^{\prime }}\leftarrow \xi \theta^{\mu }+(1-\xi )\theta^{\mu^{\prime }}\;\;\theta^{Q^{\prime }}\leftarrow \xi \theta^{Q}+(1-\xi )\theta^{Q^{\prime }}$
    end  end

## 5. Simulation Results

We primarily validate the effectiveness of the OTDDPG algorithm through numerical simulations. We assessed the method’s performance across various scenarios and compared it with other baseline methods. We conducted the simulation design using Python, and the simulation parameters are presented in Table 2.

We considered a 1 km×1 km range for UAV power inspection, with a multi-antenna base station integrated with the MEC server located at the center. We deployed N=4 UAVs to perform power inspection tasks, with a flight height H=100 m, flight time τ=1 s, maximum flight speed $v_{max}=10\;m/s$, and UAV mass M=9.65 Kg. The entire system time T=500 s was divided into I=50 time slots. The distance of a 1 m channel gain was $\beta_{0}=-50\;\mathrm{dB}$. The channel bandwidth allocated to each UAV was $B_{0}=1\;\mathrm{MHz}$. The transmission power of the UAVs was $P_{uav}=1\;w$. The noise power was $\sigma^{2}=-100\;\mathrm{dBm}$. The transmission loss was $P_{NLOS}=20\;\mathrm{dB}$. The UAV battery capacity was E=374 KJ. The UAVs’ CPU computing frequency was $f_{uav}=1.2\;\mathrm{GHz}$ and the MEC server operated at $f_{mec}=5\;\mathrm{GHz}$. The number of CPU cycles required per byte of data was s=1000 cycles/bit. The energy coefficient of the UAV’s chip architecture was $k_{uav}=10^{-28}$.

The Critic and target Critic network structures are identical, with every neuron in the upper layer establishing complete connections with the neurons in the lower layer. The input layer includes neurons for system state input and N UAV action input neurons. The neural network comprises two hidden layers with 256 and 128 neurons each. Due to the non-linear nature and computational simplicity of ReLU, it can quickly learn features of the data, accelerating the convergence process. Therefore, the ReLU activation function was employed. The output layer consists of one neuron, outputting the Q-value for state–action pairs.

The Actor and target Actor network architectures closely resemble the structure of the Critic network. A distinction arises from the Actor network’s input layer, which is determined by the dimension of the system state space. Additionally, the output layer consists of N neurons, using the tanh activation function to output the task offloading rates for N UAVs. The size of the experience replay buffer was 10,000, with a batch size of 128 samples. The discount factor was γ = 0.9. Gaussian noise follows a normal distribution with a mean of 0 and a variance of 0.05. The soft update coefficient was ξ=0.001. The learning rates for the Actor and Critic networks were 0.001 each. The number of training episodes was 1000, and the optimizer used was Adaptive Moment Estimation (Adam). The network training and updating process follows Algorithm 1.

**Figure 3:**
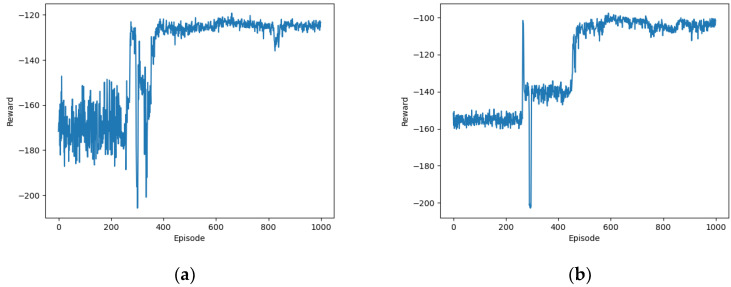
Impact of Gaussian noise on the performance of the proposed algorithm: (**a**) not considering noise; (**b**) considering noise.

Figure 3 illustrates the impact of Gaussian noise on the convergence performance of the proposed OTDDPG algorithm. It can be observed that without behavioral noise, the algorithm exhibited significant fluctuations in the first 400 episodes, resembling a difficulty in exploring optimal behavioral decisions. The reward values stabilized around −125 after 400 episodes. The convergence performance of the OTDDPG algorithm with behavioral noise was noticeably superior to that without behavioral noise. The reward values converged around −100 after nearly 500 episodes of training. This is because the role of behavioral noise during training is to introduce a certain degree of exploration, making it more likely for the agent to discover new and better strategies.

**Figure 4:**
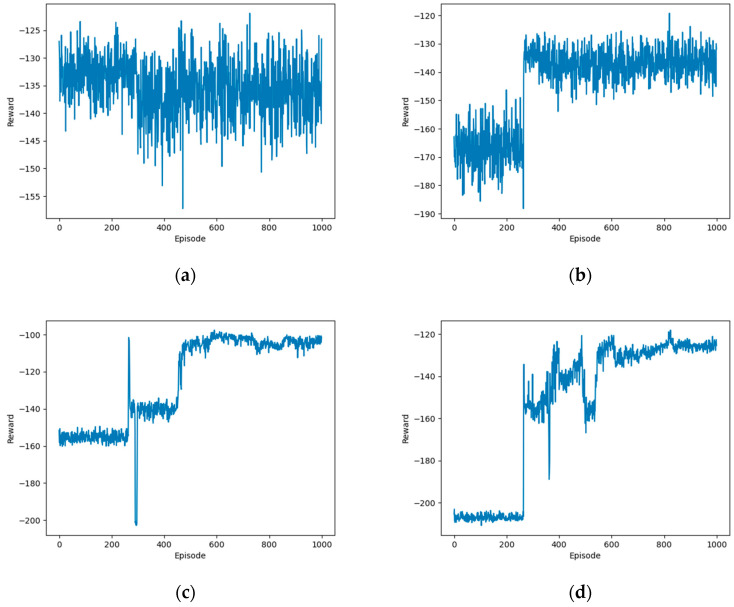
Performance comparison of the proposed algorithm under different Gaussian noise variances: (**a**) $\sigma_{e}=0.005$; (**b**) $\sigma_{e}=0.01$; (**c**) $\sigma_{e}=0.05$; (**d**) $\sigma_{e}=0.1$.

Figure 4 presents the performance comparison of the proposed OTDDPG algorithm under different Gaussian noise variances. Gaussian noise is often employed in the exploration strategy of OTDDPG algorithms. By adding noise to the actions outputted by the deterministic policy, randomness is introduced, thus aiding the agent in exploring the environment. The variance of the noise determines the level of exploration: a smaller variance leads to less exploration, while a larger variance results in more extensive exploration. When $\sigma_{e}=0.005$ and $\sigma_{e}=0.01$, which are smaller noise variances, the exploration space of the OTDDPG algorithm is limited, making it difficult for the agent to escape from local optima and potentially preventing it from discovering the global optimal strategy due to a lack of diversity when dealing with complex environments. As a result of insufficient exploration, the algorithm exhibited significant fluctuations and instability in reward values during the convergence process. When $\sigma_{e}=0.1$, this larger noise variance provided a broader exploration space for the agent, allowing it to access more diverse state–action pairs. However, this extensive exploration can also lead to an increase in convergence time, as the agent requires more time to stabilize its strategy and learn to extract useful information from a wide range of experiences. The reward value stabilizing at around −130 after nearly 800 episodes indicates that, although the algorithm may eventually converge to a relatively good strategy, the convergence process is relatively slow. When $\sigma_{e}=0.05$, a moderate level of noise variance, the algorithm seemed to find a balance, where it has sufficient exploration space to avoid local optima without slowing down convergence due to excessive exploration. The reward value stabilizing at around −100 after approximately 500 episodes of training suggests this value as a favorable Gaussian noise variance for the proposed algorithm.

**Figure 5:**
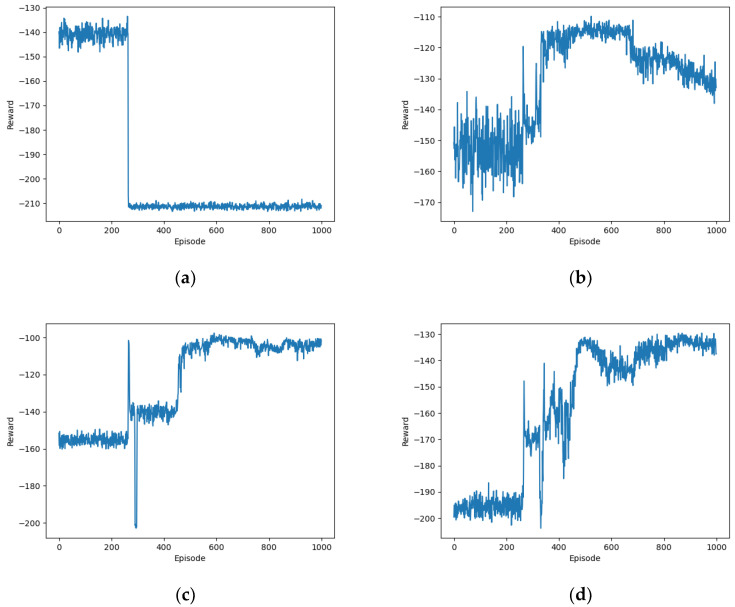
Performance comparison of the proposed algorithm under different learning rates: (**a**) $\alpha_{actor}=0.1$ and $\alpha_{critic}=0.1$; (**b**) $\alpha_{actor}=0.01$ and $\alpha_{critic}=0.01$; (**c**) $\alpha_{actor}=0.001\;and\;\alpha_{critic}=0.001$; (**d**) $\alpha_{actor}=0.0001$ and $\alpha_{critic}=0.0001$.

Figure 5 depicts the convergence performance of the proposed OTDDPG algorithm under different learning rates. When $\alpha_{actor}=0.1$ and $\alpha_{critic}=0.1$, the algorithm crashed after approximately 260 episodes of training. This was due to the excessively high learning rate causing the issue of gradient explosion. The oversized gradient updates led to an overflow of weight values, resulting in numerical instability and ultimately causing the training to crash. Similarly, when $\alpha_{actor}=0.01$ and $\alpha_{critic}=0.01$, which are relatively high learning rates, the reward value exhibited a continuous oscillation. Although it stabilized after 400 episodes, it started to decrease after 620 episodes. This suggests that the learning rate may still be too high, leading to instability in the optimization process of the algorithm. The continuous oscillation was due to the excessively large weight updates of the policy and value networks at each step, preventing the algorithm from stably converging to a good strategy. When $\alpha_{actor}=0.0001$ and $\alpha_{critic}=0.0001$, which are very low learning rates, the algorithm struggled to converge. After approximately 800 training iterations, the reward value stabilized at around −135. This was because the parameter update speed of the deep neural network (DNN) became very slow, requiring more iterations to achieve convergence. Although lower learning rates can reduce the risk of gradient explosion, they may also lead to a very slow training process and even potentially trap the algorithm in local optima. When $\alpha_{actor}=0.001$ and $\alpha_{critic}=0.001$, which are moderate learning rates, the reward value exhibited small oscillations and converged to around −100 after nearly 500 training iterations. This suggests that this is the optimal learning rate for the proposed algorithm. Moderate learning rates allow the algorithm to strike a balance between exploration and exploitation. They are not too large, which could lead to instability during training, nor are they too small, which could result in a slow training process or getting trapped in local optima. This suggest that $\alpha_{actor}=0.001$ and $\alpha_{critic}=0.001$ are the optimal learning rates for the proposed algorithm.

**Figure 6:**
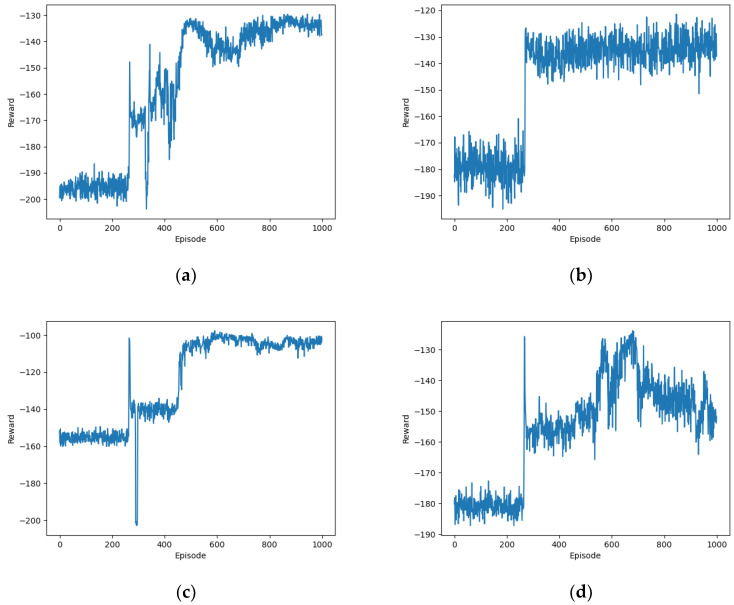
Performance comparison of the proposed algorithm under different discount factors: (**a**) γ=0.1; (**b**) γ=0.5; (**c**) γ=0.9; (**d**) γ=0.999.

Figure 6 illustrates the convergence behavior of the proposed OTDDPG algorithm under different discount factors. In reinforcement learning, the discount factor γ has a value between 0 and 1 that balances the importance of immediate rewards and long-term rewards. When γ is close to 0, the agent prioritizes immediate rewards; when γ is close to 1, the agent prioritizes long-term rewards. The discount factor plays a crucial role in both value iteration and policy iteration, affecting the calculation of Q-value functions and V-value functions, which in turn, affect the decision-making process of the agent. When γ=0.1 and γ=0.5, which are smaller discount factors, the algorithm exhibited poor convergence, resulting in significant fluctuations in reward values between −130 and −140. This was because the agent prioritized immediate rewards, potentially leading to an overemphasis on current tasks while neglecting long-term goals. An excessively small discount factor can cause the agent to engage in shortsighted behavior, preventing it from learning effective long-term strategies. When γ=0.999, a discount factor close to 1, the reward values exhibited relatively small fluctuations and tended to stabilize within the first 500 episodes. However, after 500 episodes, there was a significant oscillation in the reward values between −160 and −130. An excessively large discount factor may cause the agent to overemphasize future rewards, making it difficult to find a balance between immediate and future rewards. This can lead to oscillations during the optimization process, challenging the convergence of the algorithm. When γ=0.9, an appropriate discount factor, the algorithm placed greater emphasis on long-term rewards, encouraging the agent to engage in more strategic and far-reaching planning and decision-making. After nearly 500 training iterations, the reward values stabilized and converged around −100. An appropriate discount factor allows the agent to find a balance between immediate and future rewards, enabling it to learn effective long-term strategies. This suggests that γ=0.9 is the optimal discount factor for the proposed algorithm.

We compared the following baseline algorithms:

Local Offloading (LO): Inspection computation tasks are all executed locally by the UAVs until the energy is depleted or all tasks are completed.

Full Offloading (FO): Inspection computation tasks are all executed by the MEC server.

Random Offloading (RO): The offloading ratio of inspection computing tasks is random.

Greedy algorithm: The algorithm is a greedy strategy-based approach that selects the optimal solution at each step based on the current state, without considering potential future scenarios.

Dynamic programming (DP): This algorithm is an optimization method based on staged decision-making, decomposing the problem into a series of subproblems and progressively solving these subproblems using the optimal substructure property, ultimately obtaining the optimal solution to the original problem.

Actor Critic (AC): The algorithm frameworks of AC and DDPG are similar, but they are adapted to different problems.

Deep Q Network (DQN): The action space of DQN is discrete, so it needs to quantize its continuous action values into finite discrete values.

**Figure 7:**
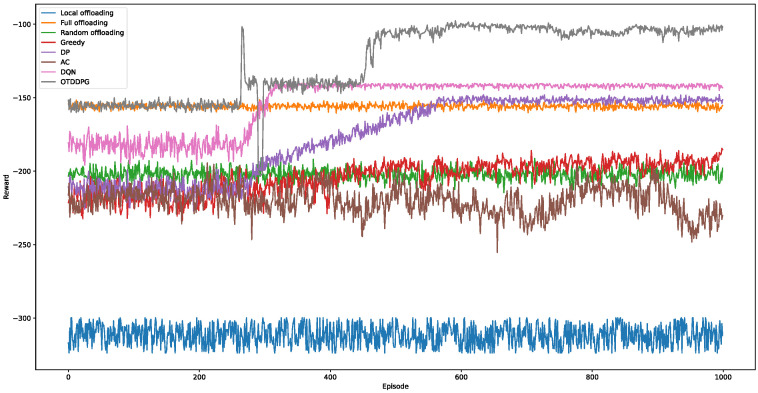
Performance comparison of different algorithms.

Figure 7 illustrates the performance comparison of the different algorithms. Each algorithm underwent 1000 episodes of training. It can be observed that the LO algorithm had the lowest reward value, with rewards falling below −300. This is attributed to all tasks being executed locally by the UAVs, resulting in substantial energy consumption and processing delay, making it the worst performing algorithm. The FO algorithm demonstrated stable performance with a reward value maintained at around −155, indicating its effectiveness in optimizing latency and overall performance. In contrast, the RO algorithm had a slightly lower reward value, hovering around −200. The main difference between the FO and RO algorithms lies in their approach to offloading tasks to edge servers, which is particularly significant in reducing latency.

The Greedy algorithm exhibited a progressive improvement trend. Initially, its rewards fluctuated significantly, showing a mediocre performance. However, between episodes 300 and 560, the Greedy algorithm began to show some improvement, although the magnitude of the improvement was not dramatic. Eventually, after round 560, the rewards of the Greedy algorithm converged to around −190. It is worth noting that in the early stages of training, the performance of the Greedy algorithm was not as good as that of the RO algorithm. However, starting from round 450, the performance of the Greedy algorithm gradually matched and even surpassed the RO algorithm in some cases. This is mainly attributed to the heuristic rules adopted by the Greedy algorithm, which select the local optimal solution as the offloading strategy at each step. However, the convergence effect of the Greedy algorithm is not ideal, which is related to its focus on local optimal solutions. The DP algorithm showed a similar performance to the Greedy algorithm in the early stages of training, with rewards experiencing an oscillation phase, and the rewards were initially lower than those of the RO algorithm. However, between episodes 270 and 570, the DP algorithm experienced a significant improvement phase. During this stage, the performance of the DP algorithm gradually surpassed that of the RO and FO algorithms, ultimately converging to fluctuations around −150. Compared to the Greedy algorithm, the DP algorithm had a longer convergence time but significantly better convergence effects. This was mainly due to the characteristic of the DP algorithm based on the optimal substructure property. By combining the optimal solutions of subproblems to construct the optimal solution of the original problem, this feature enables it to find better solutions when dealing with complex problems. However, the computational complexity of this approach is relatively high. 

On the other hand, the performance of the AC algorithm was poor, with rewards severely oscillating between −250 and −200. This instability mainly stemmed from the competitive relationship between the policy and value functions during training, making it difficult for the algorithm to converge. This competition seems to prevent the AC algorithm from effectively learning task offloading strategies, thereby affecting its performance. The performance of the DQN algorithm was second only to OTDDPG. Initially, the rewards of the DQN algorithm oscillated around −180, showing some instability. However, between episodes 265 and 325, the rewards of the DQN algorithm experienced a significant improvement, ultimately converging to fluctuations around −140. This improvement is mainly attributed to the exploration ability of the DQN algorithm in the action space, enabling it to gradually determine the optimal task offloading strategy.

The proposed OTDDPG algorithm outperformed the other seven algorithms, achieving the lowest energy consumption and latency. During training, we observed a slight improvement in rewards around round 270, stabilizing around −140. This indicates a performance improvement as the OTDDPG algorithm continuously learned and adapted to the environment. However, between episodes 270 and 450, the algorithm entered a brief convergence state. The learning bottleneck during this stage led to the algorithm being trapped in a local optimum, making it difficult to further enhance performance. Nevertheless, between episodes 450 and 480, the rewards of the OTDDPG algorithm experienced another improvement, converging to fluctuations around −100. This breakthrough improvement is attributed to further exploration and learning during longer-term training. Through continuous adjustment and optimization of policies, the OTDDPG algorithm ultimately achieved an approach close to the ideal operational strategy, providing an efficient solution to the task offloading problem.

## 6. Conclusions

This paper proposed a DRL-based task offloading optimization approach, aiming to devise an efficient, real-time, and adaptable strategy for task offloading in a UAV inspection system. An innovative UAV-Edge server collaborative computing architecture was constructed. Based on the UAV’s operational mode, we constructed a computational model for energy consumption and latency. By proposing the OTDDPG algorithm, we formulated the task offloading problem as an MDP with the aim of optimizing the objective function to minimize the weighted sum of energy consumption and latency. The simulation results demonstrated that the designed DRL network architecture exhibits advantages such as fast response, smooth stability, good generalization, and strong robustness, enabling it to autonomously adapt and learn in unknown environments.

## Figures and Tables

**Table 1:** The symbols for the variables.

Variable	Meaning
$Q_{mec}$	The fixed position of the MEC sever
$Q_{uav,k}$	The position of the k-th UAV
$h_{k}(i)$	The channel gain between the k-th UAV and the MEC server
$r_{k}(i)$	The uplink transmission rate of the k-th UAV
$c_{k}(i)$	The symbol of obstruction
$R_{k}(i)$	The task proportion of the k-th UAV
$D_{k}(i)$	The size of the data used for computations for the k-th UAV
$t_{uav,k}(i)$	The computation delay of the k-th UAV
$t_{tr,k}(i)$	The transmission delay of the k-th UAV
$t_{mec,k}(i)$	The computation delay of the MEC server for the k-th UAV
$t_{total}(i)$	The maximum task processing delay
$e_{fly,k}(i)$	The flying energy consumption of the k-th UAV
$e_{uav,k}(i)$	The computational energy consumption of the k-th UAV
$e_{tr,k}(i)$	The transmission energy consumption of the k-th UAV
$e_{total}(i)$	The total energy consumption

**Table 2:** Simulation parameters.

Parameter	Value
N	4
H	100 m
τ	1 s
$v_{max}$	10 m/s
M	9.65 Kg
T	500 s
I	50
$\beta_{0}$	−50 dB
$B_{0}$	1 MHz
$P_{uav}$	1 w
$\sigma^{2}$	−100 dBm
$P_{NLOS}$	20 dB
E	374 KJ
$f_{uav}$	1.2 GHz
$f_{mec}$	5 GHz
s	1000 cycles/bit
$k_{uav}$	$10^{-28}$

## Data Availability

Data are contained within the article.
